# Systemic Lupus Erythematosus Presenting as Isolated Thrombocytopenia: A Case Report and Review of the Literature

**DOI:** 10.7759/cureus.68354

**Published:** 2024-08-31

**Authors:** Sophia Tahir, Naufin N Ashraf, Rizwanullah, Hashem Elessawy, Sohail Khan, Samah Badr Hamad, Waqar Khan

**Affiliations:** 1 Internal Medicine, Windsor University School of Medicine, Basseterre, KNA; 2 Internal Medicine, Hayatabad Medical Complex Peshawar, Peshawar, PAK; 3 Medicine and Surgery, New York Institute of Technology College of Osteopathic Medicine, New York, USA; 4 Immunology, University of Khartoum, Khartoum, SDN

**Keywords:** pakistan, idiopathic immune thrombocytopenia, isolated thrombocytopenia, immune thrombocytopenia, systemic lupus erythematosus

## Abstract

Immune thrombocytopenic purpura (ITP) is an autoimmune disorder characterized by a low platelet count, which can lead to increased bleeding and bruising. Systemic lupus erythematosus (SLE) is a chronic autoimmune disease that can affect multiple organ systems and often presents with various hematologic abnormalities, including thrombocytopenia. A 32-year-old woman presented to the emergency department with petechiae, extensive ecchymosis, rectal bleeding, generalized body aches, anorexia, and weakness. Despite showing no clinical features of SLE, laboratory findings revealed severe thrombocytopenia and anemia. Initial treatment with low-dose steroids showed no improvement, but a high-dose steroid regimen significantly increased her platelet count. Further investigations revealed elevated ANA and positive anti-dsDNA, leading to a diagnosis of isolated thrombocytopenia as the initial manifestation of SLE. The subsequent findings of elevated ANA and positive anti-dsDNA confirmed the diagnosis of SLE, with ITP as its initial manifestation. This case underscores the importance of considering underlying autoimmune diseases in patients presenting with isolated thrombocytopenia after ruling out other causes. Early recognition and appropriate treatment of autoimmune conditions like SLE can significantly improve patient outcomes, even when initial presentations are atypical.

## Introduction

Thrombotic thrombocytopenic purpura (TTP) and immune thrombocytopenic purpura (ITP) can be initial presenting symptoms of systemic lupus erythematosus (SLE), potentially occurring months or years before full-blown SLE develops [1]. Unlike primary ITP, secondary ITP usually presents with mild to moderate thrombocytopenia. Antiplatelet antibodies are present in 78% of SLE patients, often without thrombocytopenia, and isolated thrombocytopenia precedes SLE in about 16% of cases [2]. Thrombocytopenia can be an initial symptom in 5%-16% of SLE patients. A recent meta-analysis found that approximately 2% of individuals with primary ITP may develop SLE, with female gender and positive antinuclear antibody (ANA) status being significant risk factors [3]. Another study estimates that 3%-15% of patients initially diagnosed with isolated ITP will later develop SLE [4]. We present a unique case of a 32-year-old woman who exhibited petechiae, ecchymosis, rectal bleeding, generalized body aches, and anorexia. Despite lacking typical SLE features, she was diagnosed with ITP as the initial manifestation of SLE.

## Case presentation

A previously healthy 32-year-old woman presented to the emergency department at Hayatabad Medical Complex with a sudden onset of symptoms over the past two days, including petechiae, extensive ecchymosis, rectal bleeding, generalized body aches, anorexia, and weakness. She denied experiencing night sweats, weight loss, rash, photosensitivity, or hematemesis. Her medical history included two months of oral contraceptive use but no history of smoking, drug abuse, or autoimmune diseases in the family.

Upon arrival, her vital signs were stable, and a physical examination revealed scattered petechiae and ecchymosis on her trunk and limbs, with no lymphadenopathy or visceromegaly. Cardiovascular, neurological, and rheumatological examinations were unremarkable. She was admitted and treated with 1 gram of intravenous tranexamic acid and fluid supplementation. Initial blood tests showed anemia and severe thrombocytopenia, which did not improve despite receiving 7 units of platelets.

Further investigations, detailed in Tables 1, 2, revealed elevated CRP and ESR levels, with negative rheumatoid factor and anti-CCP antibody tests. The reticulocyte count was slightly elevated, and her coagulation profile was normal. Blood smear analysis showed no schistocytes, while bone marrow examination indicated adequate cellularity with peripheral destruction and iron deficiency anemia. H. pylori antigen was positive, but tests for malaria and dengue were negative. Imaging studies, including abdominal and pelvic ultrasound, chest X-ray, and echocardiogram, revealed no abnormalities.

**Table 1:** Laboratory investigations during hospital admission mEq/L, milliequivalents per liter; mg/dL, milligram per deciliter; IU/mL, international units per milliliter; mm/1st hour, millimeters per hour; BUN, blood urea nitrogen; ALP, alkaline phosphatase; ALT, alanine transaminase; RBS, random blood sugar; ESR, erythrocyte sedimentation rate; CRP, C-reactive protein; RF, R factor; anti-CCP, anti cyclic citrullinated peptide.

Investigation	Normal range	Values
Sodium (mEq/L)	135–145	141
Chloride (mEq/L)	96–112	108
Potassium (mEq/L)	3.5–5.3	4.1
BUN (mg/dL)	18–46	21
Creatinine (mg/dL)	0.3–1.1	0.8
Retic count (%)	0.5–2.5	2.9
ALP (mg/dL)	40–145	76
Total bilirubin (mg/dL)	0.1–1.2	0.7
ALT (IU/L)	10–50	45
RBS (mg/dL)	70–140	87
ESR (mm/1^st^ hr)	0–20	47
CRP (mg/dL)	<0.5	1.5
Direct Coombs	–	Positive
Indirect Coombs	–	Negative
RF	–	Negative
Anti-CCP	–	Negative
Malarial parasite	–	Negative
Dengue IgG and IgM	–	Negative
Anti-nuclear antibody	<1:80	1:1280 Homogenous
Anti-dsDNA	<1.5	4.5
Anti-Jo-1	<1.5	Negative
Anti-SS-B (La)	<1.5	Negative
Ant-SS-A (Ro)	<1.5	Negative
Anti-Sm	<1.5	Negative
Anti-Scl-70	<1.5	Negative
Anti-RNP	<1.5	Negative
Anti-phospholipid antibody	<1.5	Negative
Complement 3 (mg/dL)	70–160	98
Complement 4 (mg/dL)	8–40	34

**Table 2:** Complete blood count during the hospital stay

Investigation	Unit (normal range)	Day 1	Day 2	Day 3	Day 4	Day 5	Day 6	Day 7	Day 8	Day 9	Day 10
Hemoglobin	g/dL (11.5–17.5)	9.1	9.5	11.4	10.9	11.4	10.7	8.7	8.9	9.6	9.5
Total leukocyte count (TLC)	×10^3 ^/µL (4–11)	9.76	10.42	11.82	6.71	5.94	8.36	11.71	10.16	12.1	1.6
Platelet count	×10^3 ^/µL (150–450 )	16	19	55	31	5	2	2	38	129	145

**Figure 1:**
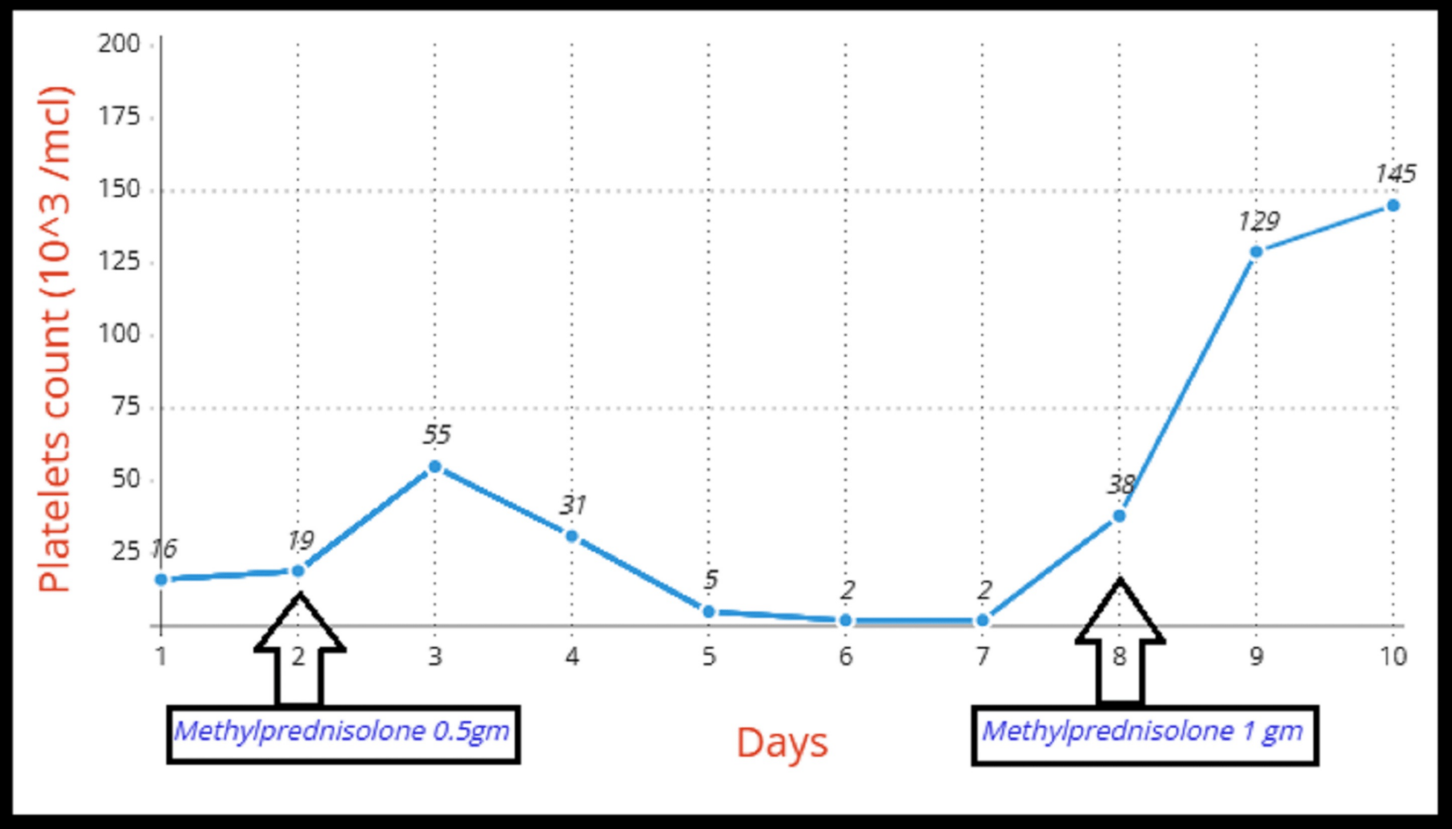
Trends in platelet counts during hospital admission

The patient was diagnosed with ITP and started on methylprednisolone 500 mg/day for 3 days, which initially improved her platelet count (Figure 1). However, the count declined after the cessation of steroids. *H. pylori *eradication therapy was also initiated. A significantly elevated homogeneous serum ANA titer (1:1280, normal <1/80), which is highly associated with SLE, was noted. Despite extensive platelet transfusions and methylprednisolone pulses, there was no significant response by the 8th day. Methylprednisolone was resumed at 1 gram/day for three days, leading to a substantial increase in platelet count to 145x10^3^/µL (Figure 1). Additionally, anti-dsDNA testing returned positive.

On the 11th day, the patient was discharged with a regimen of oral hydroxychloroquine 400 mg/day and oral prednisolone 1.5 mg/kg/day. Three weeks later, her platelet count normalized, and she remained symptom-free. Based on her clinical presentation, positive response to high-dose steroids, elevated homogeneous ANA titer, and positive anti-dsDNA, she was diagnosed with SLE presenting as isolated thrombocytopenia. Close monitoring for potential additional SLE symptoms was advised.

## Discussion

In this case, a 32-year-old woman presented to the emergency department with symptoms including petechiae, extensive ecchymosis, rectal bleeding, generalized body aches, anorexia, and weakness. Although she did not initially exhibit clinical features commonly associated with SLE, her laboratory results indicated severe thrombocytopenia and anemia. The patient did not respond to initial treatment with low-dose steroids; however, a regimen of high-dose steroids resulted in a significant increase in her platelet count. Further diagnostic workup revealed elevated ANA and positive anti-dsDNA antibodies, leading to the diagnosis of isolated thrombocytopenia as the initial manifestation of SLE.

The exact mechanism linking SLE development in ITP patients remains unclear. ITP can arise from two primary causes: insufficient platelet production due to disrupted megakaryocyte maturation or excessive antibody-mediated platelet destruction that outpaces the bone marrow's ability to compensate. In autoimmune-related ITP, the immune system mistakenly targets glycoproteins on the platelet surface, leading to an autoimmune attack. The Fc portion of these autoantibodies attaches to Fc gamma receptors on macrophages, triggering platelet phagocytosis. Notably, studies have shown that SLE patients with thrombocytopenia have a higher prevalence of antibodies against platelets compared to those with normal platelet counts. Additionally, antibodies against the antithrombopoietin receptor are more common in these patients [5].

ITP can evolve into SLE in 3%-16% of cases [6]. One study found that 12.8% of SLE cases had ITP as the only initial manifestation [7]. A long-term study by Hazzan et al. revealed that 3.6% of 222 ITP patients eventually developed SLE, with most being females who tested positive for ANA [8]. Conversely, a study by Altintas et al. reported no progression to SLE in ANA-positive children with ITP [9]. Although severe bleeding due to thrombocytopenia is rare, a case-control study indicated that SLE patients with thrombocytopenia experienced more significant organ damage, suggesting a more active form of the disease. Thus, early identification of ITP patients at risk of developing SLE is crucial for prognosis and treatment [10-12].

To predict SLE development in ITP patients, researchers have sought clinical and laboratory markers. For instance, one study found that 20% of 117 adults with ITP had positive ANA titers, and 17% of these patients (all women with high ANA titers) later developed SLE [13]. Similarly, another study involving 82 chronic ITP patients reported that 20% were ANA-positive, with 57% of these patients developing SLE either at presentation or shortly thereafter [14]. However, it's worth noting that 29 chronic ITP patients with positive ANA results did not develop SLE over a three-year period, suggesting that ANA positivity alone may not reliably predict SLE development in ITP patients [15]. Consequently, further research is necessary to fully understand the potential for SLE development in this population.

Thrombocytopenia is common in SLE, complicating the distinction between idiopathic and secondary ITP. The condition may result from underlying diseases or therapeutic interventions. Secondary ITP has a more complex pathophysiology, necessitating a thorough diagnostic approach, particularly in patients on steroids or immunosuppressive medications [16]. In patients with thrombocytopenia who meet the criteria for SLE, SLE-associated ITP can be diagnosed after excluding other causes such as drug effects, splenomegaly, antiphospholipid syndrome, or thrombotic microangiopathy. A comprehensive clinical and laboratory evaluation, including a full blood count, peripheral smear, and screening for antiphospholipid syndrome, is essential to determine the cause of thrombocytopenia in SLE. It is also crucial to exclude pharmacologically induced thrombocytopenia, which presents a significant diagnostic challenge.

The management of SLE with thrombocytopenia closely mirrors that of ITP, with treatment strategies tailored to the underlying mechanisms. Initial treatments typically include IV immunoglobulin (IVIG), steroids, and anti-D IG. If first-line treatments fail, second-line therapies such as thrombopoietin receptor agonists (TPO-RA) and rituximab may be considered. TPO-RA options include romiplostim, eltrombopag, and avatrombopag [17]. For cases unresponsive to second-line treatments, third-line therapies such as azathioprine, cyclosporine A, decitabine (low doses), fostamatinib, hydroxychloroquine, mycophenolate mofetil, oseltamivir, and tacrolimus may be utilized [18, 19].

A study of 59 SLE patients with thrombocytopenia revealed that 40 responded positively to steroid therapy with increased platelet counts, though only 11 maintained this response over an average follow-up of 78 months. High-dose intravenous methylprednisolone initially benefited 60% of patients, but none sustained the response long-term [19]. In another study involving 53 cases, high-dose steroids were followed by cyclophosphamide or azathioprine, with or without IVIG. While all patients achieved a normal platelet count, 44% experienced relapses during the disease course [20].

In our case, the patient did not respond to low-dose steroids. However, after confirming ANA positivity, we diagnosed the condition as secondary ITP associated with SLE. Increasing the steroid dosage led to significant improvement, highlighting the necessity of high-dose steroids in treating ITP secondary to SLE. This case was particularly challenging because the patient exhibited no other features of SLE, underscoring the importance of considering underlying autoimmune diseases in patients presenting with isolated thrombocytopenia, especially after ruling out other causes.

## Conclusions

In conclusion, the case presented underscores the complexity and diagnostic challenges associated with thrombocytopenia, particularly when it manifests as an initial symptom of SLE. The overlap between ITP and SLE highlights the importance of thorough clinical evaluation and appropriate laboratory investigations to identify the primary and secondary causes of thrombocytopenia. High-dose steroid therapy was crucial in achieving a favorable response in this patient, emphasizing the therapeutic considerations in managing ITP secondary to SLE. Clinicians should maintain a high index of suspicion for underlying autoimmune conditions, such as SLE, in patients presenting with isolated thrombocytopenia, even in the absence of typical SLE symptoms. A limitation of our study is the short follow-up period; a case with long-term follow-up would better reflect the real scenario. Further research is needed to elucidate the precise mechanisms linking these conditions and to refine diagnostic and treatment strategies for better patient outcomes.
